# Modified Vaccinia Virus Ankara (MVA) as Production Platform for Vaccines against Influenza and Other Viral Respiratory Diseases

**DOI:** 10.3390/v6072735

**Published:** 2014-07-17

**Authors:** Arwen F. Altenburg, Joost H. C. M. Kreijtz, Rory D. de Vries, Fei Song, Robert Fux, Guus F. Rimmelzwaan, Gerd Sutter, Asisa Volz

**Affiliations:** 1 Department of Viroscience, Erasmus Medical Center (EMC), P.O. Box 2040, 3000 CA Rotterdam, The Netherlands; E-Mails: a.altenburg@erasmusmc.nl (A.F.A.); j.kreijtz@erasmusmc.nl (J.H.C.M.K.); r.d.devries@erasmusmc.nl (R.D.V.); 2 Institute for Infectious Diseases and Zoonoses, LMU, University of Munich, 80539, Munich, Germany; E-Mails: fei.song@micro.vetmed.uni-muenchen.de (F.S.); robert.fux@micro.vetmed.uni-muenchen.de (R.F.); asisa.volz@micro.vetmed.uni-muenchen.de (A.V.)

**Keywords:** modified vaccinia virus ankara, vaccine development, influenza virus, respiratory syncytial virus, parainfluenza virus, coronavirus

## Abstract

Respiratory viruses infections caused by influenza viruses, human parainfluenza virus (hPIV), respiratory syncytial virus (RSV) and coronaviruses are an eminent threat for public health. Currently, there are no licensed vaccines available for hPIV, RSV and coronaviruses, and the available seasonal influenza vaccines have considerable limitations. With regard to pandemic preparedness, it is important that procedures are in place to respond rapidly and produce tailor made vaccines against these respiratory viruses on short notice. Moreover, especially for influenza there is great need for the development of a universal vaccine that induces broad protective immunity against influenza viruses of various subtypes. Modified Vaccinia Virus Ankara (MVA) is a replication-deficient viral vector that holds great promise as a vaccine platform. MVA can encode one or more foreign antigens and thus functions as a multivalent vaccine. The vector can be used at biosafety level 1, has intrinsic adjuvant capacities and induces humoral and cellular immune responses. However, there are some practical and regulatory issues that need to be addressed in order to develop MVA-based vaccines on short notice at the verge of a pandemic. In this review, we discuss promising novel influenza virus vaccine targets and the use of MVA for vaccine development against various respiratory viruses.

## 1. Introduction

Respiratory viruses, such as seasonal and pandemic influenza viruses, human parainfluenza virus (hPIV), respiratory syncytial virus (RSV) and coronaviruses, cause substantial burden of disease globally. These pathogens cause respiratory tract infections, mainly in young children, the elderly and immunocompromised individuals. In contrast to seasonal influenza, currently no licensed RSV and hPIV vaccines are available. 

For influenza, it is recommended to annually vaccinate people at risk to protect them against infection with seasonal influenza viruses. However, as a result of selective pressure exerted by virus‑specific antibodies induced by previous infections and/or vaccination, seasonal influenza viruses accumulate mutations in the antigenic sites of the two main surface proteins: hemagglutinin (HA) and neuraminidase (NA). Consequently, antigenic drift variants emerge that evade host immunity.

Occasionally, avian or swine influenza viruses are introduced into the human population. Since neutralizing antibodies to these novel viruses are virtually absent, the human population at large is susceptible to infection. Last year alone, several avian influenza viruses caused human infections. From February 2013 to February 2014, 335 human cases of infection with H7N9, of which some viruses display signs of adaptation to humans, have been reported [1,2]. One hundred and twelve of these cases had a fatal outcome [1]. Although sustained human-to-human transmission of these viruses has not been reported, it is possible that they acquire this ability with just a few mutations as was shown experimentally for H5N1 viruses in ferrets [3]. In addition, human cases of infections with avian viruses of the H10N8 and H9N2 subtype have been reported, some with a fatal outcome [1]. If one of these viruses becomes transmissible from human-to-human, it can cause a widespread outbreak that could evolve into an influenza pandemic with considerable morbidity and mortality. 

In terms of pandemic preparedness, procedures should be in place to respond rapidly and produce tailor made vaccines on short notice. Furthermore, there is a need for the development of universal influenza vaccines that induce broad protective immunity against human influenza viruses and potentially pandemic viruses of various subtypes. 

## 2. Targets for Influenza Vaccine Development

### 2.1. Conventional Influenza Vaccine

Currently, trivalent inactivated vaccines are most commonly used to protect risk groups against seasonal influenza virus infection. These vaccines contain components of three virus strains responsible for epidemic outbreaks: H1N1 and H3N2 influenza A viruses and an influenza B virus [1,4]. Recently, quadrivalent vaccines have become available that contain an additional, antigenically different influenza B virus component [1]. These vaccines aim at the induction of virus-neutralizing antibodies against HA and NA. 

In order for the vaccines to be effective, it is of great importance that the vaccine strains antigenically match the epidemic strains. Therefore, the World Health Organization makes a recommendation for vaccine strains twice a year based on the strains that are most likely to circulate next season [1]. Using these viruses, whole inactivated vaccines, split virion vaccines or subunit vaccines are prepared. In addition to these inactivated influenza vaccines, also live-attenuated vaccines are available [4].

For decades, the use of seasonal influenza vaccines has helped to reduce influenza-related morbidity and mortality [5]. However, the preparation and use of current inactivated influenza vaccines has some limitations. First, if the vaccine strains do not match the epidemic influenza strains antigenically, vaccine effectiveness will be reduced. In addition, the seasonal influenza vaccine will offer little or no protection against influenza viruses of a novel subtype with pandemic potential. Second, the vaccine production capacity, even of all manufacturers combined, is limited. Especially in the case of a pandemic outbreak, the vaccine needs to become globally available in a short period of time [6]. Third, it takes too long for vaccines to become available using conventional production methods. For example, during the 2009 H1N1 pandemic outbreak it took six months before vaccination campaigns started in most countries, often after the peak of the pandemic [7]. Fourth, subjects in the high-risk groups may not respond optimally to vaccination and thus the vaccine is least effective in the people who need it most. Finally, inactivated influenza vaccines inefficiently induce virus-specific CD8^+^ T cells, which contribute to cross-protective immunity [8,9]. 

These limitations underscore the need for the development of novel vaccine production platforms and novel vaccine candidates that not only allow for rapid availability of vaccines in the face of an emerging pandemic, but that also may be used for the induction of broad protective immunity. Several approaches, at different stages of development, are under investigation in order to achieve the ultimate goal: a universal influenza vaccine. Here, we describe how the immunogenic potential of the different influenza virus antigens is assessed in the context of universal influenza vaccine development.

### 2.2. Induction of HA Stalk-Specific Antibodies

The antibody response induced by influenza virus infection or vaccination is mainly directed against the surface protein HA. These antibodies can have neutralizing activity by preventing virus attachment to the host cell or the post attachment fusion event. HA consists of two domains: a globular head-domain that is highly variable within and between subtypes and a more conserved stalk-domain. Based on phylogenetic analysis of the nucleotide sequence of HA, influenza A viruses can be divided in group 1 (H1, H2, H5, H6, H8, H9, H11, H12, H13, H16, H17) and group 2 (H3, H4, H7, H10, H14, H15) viruses [10].

Broadly neutralizing antibodies specific for the head-domain have been described [11,12,13,14,15,16]. However, considering the high mutation rate of this domain, eliciting an antibody response specific for the stalk-domain has more potential to induce heterosubtypic immunity. HA stalk-specific antibodies are induced after influenza virus infection or vaccination [11,17,18,19,20]. However, the magnitude of the stalk-specific antibody response varies considerably between individuals. Moreover, given the low frequency of stalk-specific B cells it is unlikely that the antibody levels induced upon influenza virus infection afford protection [11,20]. 

It has been shown that passive immunization with stalk-specific antibodies affords protection against infection with a heterologous influenza virus in mice and ferrets [11,15,18,21,22,23,24,25,26] (reviewed in [10]). Several HA stalk-based vaccine strategies have been described, including sequential vaccination with a chimeric HA that contains a conserved stalk but a head-domain of different influenza subtypes [20,26,27,28,29,30]. Furthermore, modification of the head-domain by the introduction of extra glycosylation sites to shield the head-domain from recognition by virus-specific B-cells in favor of an antibody response to the stalk-domain [31] also seems a promising strategy to induce a robust anti-stalk antibody response. However, stalk-reactive antibodies specific for both group 1 and group 2 HA-expressing influenza A viruses are rare [32]. Therefore, a universal vaccine that induces or boosts a stalk-specific antibody response would probably require components of both group 1 and group 2 HA proteins and influenza B viruses.

### 2.3. Antibody Response against NA and M2

The second surface protein NA enables the release of progeny virions from the host’s cell surface. Upon an influenza virus infection, NA-specific antibodies are induced, which can be boosted by vaccination with trivalent influenza vaccines [33]. NA-specific antibodies are not able to exert heterosubtypic immunity to the extent of HA stalk-specific antibodies. Nevertheless, it has been shown that anti-NA antibodies can provide some intrasubtypic immunity [34]. In contrast to HA, these antibodies do not prevent virus infection but rather hamper release of newly formed virus particles [35].

Not only NA-specific antibodies elicited through natural infection, but also NA antibodies induced by immunization can provide intrasubtypic protection. Vaccination with a DNA plasmid expressing NA has been shown to provide protection against infection with a structurally similar influenza virus [36,37]. Given the narrow range of protection of this NA-specific antibody response, a stand‑alone NA-based vaccine would not be the most attractive candidate for universal influenza vaccine development. However, the addition of NA to an HA component can improve the virus‑specific antibody response [38].

The third and minor surface protein is matrix protein 2 (M2), which forms ion channels in the viral envelope. M2, more specifically the M2 ectodomain (M2e), is considered a good candidate for universal influenza vaccine development because it is relatively conserved among influenza A viruses [39]. Antibodies specific for M2 are unable to neutralize the virus due to their inability to bind the protein on the virion surface. However, antibodies can bind to M2 when it is exposed on the surface of infected host cells. These antibodies mediate killing of the infected cells by antibody‑dependent cellular cytotoxicity (ADCC), most likely by natural killer (NK) cells [40]. M2‑specific antibodies may also opsonize infected cells for phagocytosis by macrophages [41,42]. 

Due to its poor immunogenicity, vaccine development based on M2 protein is challenging. However, if the M2-based vaccine is adjuvanted, a robust antibody response can be induced [43]. Several M2-based influenza vaccine candidates have been described and validated in various animal models, including DNA constructs [44], virus-like particles (VLPs) [40,41,42,45] and viral vectors [46]. It has been shown that M2-based vaccines can provide protection against infection with a heterologous virus [41,46,47]. Moreover, even six months after vaccination mice were protected from a homologous challenge infection [41], indicating that an M2-based vaccine can provide long-term protection. Currently, M2-based vaccines are tested in clinical trials [48]. However, M2-specific antibodies alone cannot provide sterile immunity [39]. Therefore, combining M2 with another influenza antigen might induce a better protective immune response [49]. 

### 2.4. Broadly Reactive T Cell Responses against Influenza Viruses

During an influenza virus infection antigen presenting cells (APCs), predominantly dendritic cells (DCs), process viral proteins into peptides for presentation to naïve T cells. Upon recognition of these peptides bound to major histocompatibility complexes (MHC), there is clonal expansion of virus‑specific CD4^+^ or CD8^+^ naïve T cells into effector cells. A second signal from a co-stimulatory molecule is required to prevent abortive clonal expansion. Activated T cells migrate to the lungs where they recognize and eliminate infected epithelial cells. When the infection is cleared, two types of memory T cells are established: long lived central memory T cells and effector memory T cells [50].

It was already recognized over30 years ago that conserved internal influenza virus proteins, like the nucleoprotein (NP) and the matrix 1 (M1) protein, are targets for CD8^+^ cytotoxic T lymphocytes (CTLs) that consequently cross-react with influenza viruses of different subtypes [51,52,53,54]. Infections with seasonal influenza virus induce CTLs that even cross-react with influenza viruses of avian or swine origin [55,56,57,58,59]. It is now generally accepted that virus-specific CD8^+^ T cells play an important role in cross-protective immunity [60]. More recently, it has been demonstrated both in animal models and humans that also CD4^+^ T helper cells contribute to cross-protective immunity [61,62,63,64,65]. Upon infection with heterologous influenza viruses, cross-reactive anamnestic T cell responses contribute to accelerated clearance of infection and reduction of clinical symptoms [66,67].

Influenza vaccines aiming at the induction of virus-specific T cells have mainly targeted the internal proteins NP and M1. These can be delivered as protein, peptide-carrier conjugate, VLP, DNA plasmid or by viral vectors [68]. However, also other relatively conserved influenza virus proteins might be considered for the induction of cross-reactive T cell responses, like the polymerase subunits (PA, PB1 and PB2).

### 2.5. Universal Influenza Vaccine

As described above, there are several promising targets for the development of a universal influenza vaccine and several vaccination strategies are being evaluated. Given that not only virus-specific antibodies but also T cells contribute to (cross-)protective immunity, it is of importance that a universal influenza vaccine activates both arms of the adaptive immune system. In this respect, the use of viral vectors for the delivery of viral proteins has advantages over conventional vaccines and holds promise. Overexpression of viral proteins potentially increases their immunogenicity. Furthermore, the use of a live vector allows *de novo* synthesis of viral proteins in the cytosol of antigen presenting cells and thus facilitates antigen processing and presentation to virus-specific CD8^+^ T cells. Alternatively, cross-priming may result in the activation of these cells. Thus, vector vaccines may not only induce virus-specific antibody responses but also induce cell-mediated immune responses. Moreover, the antigens of interest are expressed in their native conformation, thus inducing antibodies of the proper specificity. Last but not least, viral vector vaccines can be designed and produced very rapidly and can be used for large-scale vaccine production, which makes them attractive vaccine candidates in the face of an emerging pandemic outbreak. 

Various vectors are tested in the context of viral vector vaccines, of which Modified Vaccinia virus Ankara (MVA), discussed in this review, and adenovirus vectors are most prominent candidates.

## 3. MVA

### 3.1. The Development of the Attenuated Vaccinia Virus Strain MVA

Modified Vaccinia virus Ankara (MVA) was derived from Chorioallantois Vaccinia virus Ankara (CVA) through serial passaging in chicken embryo fibroblasts (CEF) [69,70]. From 1968–1985, the Bavarian State Vaccine Institute produced MVA as a human smallpox vaccine. The application of this MVA vaccine was successful to increase the safety of the conventional smallpox vaccination as documented by the absence of any serious adverse event in large field trials involving more than 120,000 individuals in Germany [71]. 

The serial passage of MVA in primary and secondary CEF cultures resulted in major deletions in the viral genome and many mutations that affected most known vaccinia virus (VACV) virulence and immune evasion factors [72,73,74]. Consequently, MVA replication is highly restricted to avian cells and the virus is unable to produce infectious progeny in most cells of mammalian origin [75,76,77]. 

### 3.2. Advantages of MVA as Viral Vector

The host cell restriction of MVA is associated with a late block in the assembly of viral particles in non-permissive cells. This phenotype is rather exceptional among poxviruses with host range deficiencies, which are usually blocked prior to this stage during the abortive infection in mammalian cells [78,79,80]. Non-replicating MVA allows for unimpaired synthesis of viral early, intermediate and abundant late gene products, which supported its development as safe and particularly efficient viral vector [77]. Moreover, the biological safety and replication deficiency of MVA has been confirmed in various *in vivo* models, including avian species and animals with severe immunodeficiencies [81,82,83,84]. Therefore, recombinant MVA viruses as genetically modified organisms can be used under conditions of biosafety level 1 in most countries, provided that innocuous heterologous gene sequences are expressed. The latter attribute is an important advantage compared to replication competent poxvirus vectors (BSL 2 organisms) and other viral vectors and has certainly contributed to the increasing use of recombinant MVA in clinical testing. 

To deliver heterologous antigens with MVA as vector vaccine, the target gene sequences are transcribed under the highly specific control of poxviral promoters that are only recognized and activated by virus encoded enzymes and transcription factors. Recombinant genes are only transiently expressed after the infection with non-replicating MVA. Since there is no survival of MVA infected host cells it can be assumed that full clearance of recombinant virus and recombinant DNA occurs within days after vaccine administration. Despite the transient production of heterologous proteins MVA vector vaccines are able to elicit high levels of antigen-specific humoral and cellular immune responses as demonstrated with the first MVA candidate vaccine delivering influenza antigens [85] (for review see [86]). It is of note that even for activation of antigen-specific CD8^+^ T cell responses, the delivery of stable proteins might be advantageous compared to immunogens that were designed for rapid intracellular degradation [87,88,89]. This seems to suggest that MVA-delivered proteins can be efficiently presented via both endogenous and cross-presentation pathways of MHC class I antigen processing (for review see [90]).

Another characteristic of MVA vaccines is their surprising level of immunogenicity and protective capacity when compared to replicating VACV vector vaccines expressing the same recombinant genes [85,91,92]. Replication competent vectors, because of their capacity to amplify *in vivo*, could be expected to infect more target cells and produce higher amounts of antigen per immunization than the non-replicating MVA vectors. Nevertheless, the efficacy of the MVA vaccinations compared favorably to the outcome of immunizations with replication competent VACV vectors in mice and non-human primates. These observations may be best explained by the capacity of MVA to readily activate various components of the host innate immune system, most probably because of the lack of many immune evasion factors encoded by wild-type VACV [93,94,95,96,97,98,99,100]. Thus, unlike other VACV strains MVA does not produce the soluble virus proteins that function as receptor-like inhibitors of type I and type II interferons, tumor necrosis factor and chemokines [93]. Moreover, MVA infection can be sensed by multiple intracellular host detection mechanisms resulting in the production of interferons, inflammatory cytokines and chemokines [95]. Here, it is noteworthy that MVA has lost several of the VACV inhibitors targeting intracellular signaling pathways, e.g., the host NF-κB activation. In consequence, *in vivo* infection with MVA but not other VACV strains can trigger the rapid immigration of monocytes, neutrophils and CD4^+^ lymphocytes to the site of inoculation [99]. These intrinsic immunostimulating activities suggest that the use of additional adjuvant systems together with MVA might be dispensable for most vaccine applications. Finally, the continuing advances in genetic engineering, process development, large-scale manufacturing and MVA-specific immune monitoring have brought recombinant MVAs into clinical trials at an increasing scale [101,102,103]. The successful development of an MVA-based next generation vaccines against smallpox licensed in Europe and Canada has also contributed to this substantial groundwork for the development of future recombinant MVA vaccines [104].

## 4. MVA as an Influenza Vaccine

As indicated above, MVA holds great promise as viral vaccine vector. It has been tested as candidate vaccine against influenza in several studies and the results of these studies are summarized here (Table 1). It has been suggested that the efficacy of vector vaccines could be hampered by the pre-existing immunity to the vector [105]. However, it has been demonstrated that with MVA this is not an issue, since with MVA, protective immunity could be induced against influenza in the presence of pre-existing vector immunity [106].

### 4.1. MVA-HA

#### 4.1.1. Vaccines against A/H5N1 Viruses

Highly pathogenic avian influenza viruses of the H5N1 subtype cause mainly endemic outbreaks in poultry. Since the first human case of infection with an avian A/H5N1 influenza virus in 1997, over 650 cases have been reported of which 386 had a fatal outcome [107]. The circulation of A/H5N1 viruses in poultry in several geographic regions continues to pose a threat to public health. A pandemic outbreak with this virus is still feared since it has been demonstrated that a handful of mutations are sufficient for these viruses to become transmissible between mammals [3,108]. The development of efficacious H5N1 vaccines is complicated by the co-circulation of viruses that belong to various antigenically distinct clades. Ideally, a novel vaccine induces antibodies that cross-react with A/H5N1 viruses from multiple clades.

Several recombinant MVA vaccines expressing an HA gene of various A/H5N1 viruses have been constructed and tested in various animal models [109]. MVA expressing the HA gene of influenza virus strain A/Vietnam/04 (MVA-HA-VN/04) induced strong antibody responses that cross-reacted with other viruses to some extent and protected mice from infection with homologous and heterologous A/H5N1 viruses [110,111]. MVA-HA-VN/04 induced superior protective immunity in mice to the homologous and heterologous H5N1 viruses compared to MVA expressing HA genes of A/H5N1 viruses A/Hong Kong/156/97, A/Indonesia/5/05, A/turkey/Turkey/1/2005, A/Chicken/Egypt/3/2006 or A/Anhui/1/2005 [111]. MVA-HA-VN/04 was also tested in non-human primates. Two immunizations with 10^8^ PFU protected cynomolgus macaques against infection with influenza viruses A/Vietnam/1194/04 and A/Indonesia/5/05 [112,113]. MVA-HA-VN/04 also proved to be immunogenic in chickens and afforded protection against infection [83]. The favorable outcome of preclinical testing of MVA-HA-VN/04 prompted the further clinical testing of this vaccine candidate in an ongoing phase I/II trial.

To assess whether protective immunity also could be achieved with lower doses of MVA, dose finding was performed in mice with MVA-HA-VN/04. Interestingly, two immunizations with a dose as low as 10^4^ PFU were sufficient to induce protective immunity against infection with homologous and heterologous viruses. However, a dose of ≥10^5^ PFU was required for the induction of sterile immunity against the homologous strain. Furthermore, a single immunization with a dose in the range of 10^5^–10^8^ PFU resulted in protection from disease, albeit no sterile immunity was achieved [114]. These data indicate that in case of a pandemic, when large numbers of vaccine doses need to be produced in a short period of time, protective immunity against H5N1 viruses can be induced with one or two low doses of MVA.

#### 4.1.2. MVA-Based Vaccines against H1N1 Viruses

In order to evaluate an MVA-based vaccine against the A/H1N1 virus that caused the pandemic of 2009, the HA gene of a prototypic strain was cloned into MVA (MVA-HA-Ca/09). Mice vaccinated with MVA-HA-Ca/09 were protected from infection with a 2009 pandemic A/H1N1 influenza virus. Protection correlated with the induction of virus neutralizing antibodies and virus-specific T cells [115]. In addition, cross-protective immunity against some but not all swine A/H1N1 influenza viruses was induced [116]. Thus, the MVA-HA-Ca/09 vaccine induces, to some extent, intrasubtypic immunity in mice.

The MVA-HA-Ca/09 vaccine was also tested in ferrets. One immunization afforded only modest protection, but a second immunization induced robust antibody titers that reduced the virus replication after challenge infection with influenza virus A/Netherlands/602/09 (H1N1pdm09). However, full sterile immunity was not achieved, which may be related to the route of administration and/or dose of challenge virus [117]. Taken together, these data indicated that an MVA-based vaccine would have been able to induce protective immunity against the virus that caused the pandemic of 2009, although the extent of cross-protection against unrelated H1N1 viruses may have been limited.

### 4.2. MVA-HA+NP

In order to elicit both virus-specific antibodies and T cell responses with a single vaccine, recombinant MVA expressing both the HA and NP genes have been constructed. MVA expressing both NP and HA genes derived from influenza virus A/Puerto Rico/8/34 induced protective antibody and CTL responses against a homologous or heterologous infection in mice [85,118]. Other MVA-HA+NP vaccines have been prepared with HA genes obtained from A/California/04/2009 (H1N1pdm09) (MVA-HA1+NP) or A/Vietnam/1203/2004 (H1N1) (MVA-HA5+NP). The use of MVA-HA1+NP induced cross-protective immunity against infection with the homologous pandemic H1N1 strain, an unrelated H1N1 strain and an H5N1 influenza virus. Furthermore, this vaccine afforded partial protection against a challenge with H3N2 influenza viruses. Thus, this recombinant MVA, expressing both the HA gene of an H1N1pdm09 virus and a highly conserved NP gene, induces heterosubtypic immunity. In contrast, MVA-H5+NP induced only protection against H5N1 virus and the pandemic H1N1 strain [106].

### 4.3. MVA-NP+M1

To design an MVA-based vaccine that aims at the induction of virus-specific T cell responses only, recombinant MVA was constructed that expresses the genes encoding the relatively conserved internal structural proteins NP and M1. The MVA-NP+M1 vaccine has been tested in phase I and phase IIa clinical trials. The vaccine induces mainly virus-specific CD8^+^ T cells, which can be detected one week after intramuscular immunization [119] and induced protective immunity against an experimental infection one month after vaccination [120]. The vaccine was not only tested in healthy adults, 18–45 years of age, but also in people over 60 years of age. It was shown that the vaccine could safely be administered to the elderly [121].

Different vaccination strategies have been investigated [122,123]. It seems that priming with an adenovirus vector expressing NP and M1 and a subsequent boost with MVA expressing the same antigens induces higher levels of protective T cells than vaccination with either of the vectors alone [123,124]. The strongest T cell responses were obtained after intramuscular administration (adenovirus vector) and intranasal or intramuscular administration (MVA) compared to intradermal injection [123]. Intramuscular immunization is preferred because the vaccine is easy to administer and there is an optimal balance between immunogenicity and side effects. In addition, combinations of priming with MVA-NP+M1 and boosting with a HA-containing component have been tested, since the MVA potentiates the second immunization [125,126]. Priming with MVA-NP-M1 and boosting with the trivalent influenza vaccine resulted in higher levels of total IgG but did not affect the number of IFNy‑producing T cells when compared to vaccination with MVA alone. The combination vaccination strategy protected mice from a heterologous challenge infection six months after immunization [125]. 

### 4.4. MVA Expressing Other Combinations of Influenza Virus Proteins

In order to develop a universal influenza vaccine, various recombinant MVAs expressing a variety of conserved antigens derived from an A/H5N1 influenza virus were constructed. These antigens include NP, PB1, M1, M2, the HA stalk-domain, HA-stalk combined with NP, HA-stalk in combination with four repeats of M2e derived from H5N1, H9N2, H7N2 and H1N1 viruses and HA-stalk+4xM2e+NP. Mice were immunized twice with the respective MVA vaccines and subsequently infected with influenza virus subtypes with pandemic potential: H5N1, H7N1 or mouse adapted H9N2. MVA expressing NP, HA-stalk+NP or HA-stalk+4xM2e+NP induced heterosubtypic immunity. Co-expression of NP seemed essential since expression of NP induced virus specific CD4^+^ and CD8^+^ T cells [127]. All the other MVA vaccines tested, MVA-HA-stalk, MVA-HA-stalk+M2e, MVA-M1, MVA-M2, MVA-PB1, failed to induce protective immunity. Interestingly, vaccination with MVA-M1 predisposed for more severe disease upon challenge infection of the mice, although the difference in survival rates with the naïve control group was not statistically significant [127]. Thus, MVA is widely used for the development of influenza vaccine, which shows encouraging results. The vector holds great promise as a vaccine platform for respiratory viruses in general.

**Table 1:** Overview of MVA-based influenza vaccines.

MVA vaccine	Response	Model	Protective efficacy after challenge	Literature	
MVA-NA-Ca/09	B cells	mice	Partial homologous protection	[115]	
MVA-HA-HK/97	B cells	mice	Homologous protection	[110]	
MVA-HA-VN/04	B cells	mice	Cross-clade protection	[106,110,111]	
		chickens	Homologous protection	[83]	
		macaques	Cross-clade protection	[38,113]	
MVA-HA-IN/05	B cells	mice	Cross-clade protection	[111]	
MVA-HA-TT/05	B cells	mice	Partial cross-clade protection	[111]	
MVA-HA-AN/05	B cells	mice	Partial cross-clade protection	[111]	
MVA-HA-CE/06	B cells	mice	Partial cross-clade protection	[111]	
MVA-HA-Ca/09	B cells	mice	Homologous protection and to some extent heterosubtypic protection against swine viruses	[115,116]	
		ferret	Intrasubtypic protection	[117]	
MVA-HAstalk	B cells	mice	No protection	[127]	
MVA-HAstalk/M2e	B cells	mice	No protection	[127]	
MVA-HAstalk/M2e+NP	B cells and T cells	mice	Heterologous protection	[127]	
MVA-HAstalk+NP	B cells and T cells	mice	Heterologous protection	[127]	
MVA-HA+NP	B cells and T cells	mice	Homologous protection	[85,106,118]	
MVA-NP	B cells and T cells	mice	Heterologous protection	[127]	
MVA-NP+M1	T cells	mice	Partial heterologous protection*	[123,125]	
		chickens	Heterologous protection*	[124,125]	
		pigs	Not tested with challenge	[125]	
		humans	Intrasubtypic protection, safe in elderly	[119,120,121,122,126]	
MVA-M1	**	mice	No protection	[127]	
MVA-M2	**	mice	No protection	[127]	
MVA-PB1		mice	No protection	[127]	
MVA-HA-Eq/Ky81 (A/Equine/Kentucky/1/81) MVA-NP-Eq/Ky81	HA: B-cells not tested	horses	HA: Homologous protectionNP: Partial homologous protectionLiterature:	[128]	

* In combination with Adenovirus vaccine. ** No protective antibody response or T cell response measured.

## 5. MVA-Based Vaccine against Other Respiratory Diseases

### 5.1. Respiratory Diseases Caused by Viruses of the Paramyxoviridae Family

Important viruses in the Paramyxoviridae family include the human parainfluenza viruses (PIV), human respiratory syncytial virus (RSV) and human metapneumovirus (hMPV). These pathogens are transmitted via the respiratory route and all are causing agents of acute respiratory tract infections in humans, particularly young children, elderly and the immunocompromised. Infections with these viruses are among the leading reasons for pediatric hospitalizations (for review see [129,130,131]). At present, there are no licensed vaccines for effective prevention of these infections, which has spurred the evaluation of first recombinant MVA vaccines against PIV and RSV. 

Recombinant MVA co-producing the fusion (F) and hemagglutinin-neuramidase (HN) proteins of PIV3 have been generated for preclinical testing in animal models [132]. In the cotton rat model, recombinant MVA elicited high levels of PIV-specific antibodies upon immunization by intramuscular or intranasal application. Upon challenge, MVA-HN was more efficient in inducing protection as determined by a substantial reduction of PIV loads in the nasal turbinates and lungs. This result favorably compared to responses achieved with an attenuated live PIV candidate vaccine. Furthermore, when used in rhesus macaques the recombinant MVA vaccines also induced protection against PIV challenge, although intranasal vaccinations could not completely prevent infections of the upper respiratory tract [133,134]. 

First generation candidate MVA vaccines against RSV expressed recombinant gene sequences encoding for either the RSV fusion protein (F) or the glycoprotein (G) or both envelope antigens together [135]. In mice, all recombinant MVA induced RSV-specific antibodies and levels of MVA vaccine induced circulating antibodies were even higher than those found after experimental RSV infection. A follow-up study with these MVA vectors in a mouse model also demonstrated the induction of strong RSV-specific T cell responses, resulting in clearance of RSV from the lungs of the vaccinated animals, although it also associated with weight-loss in vaccinated animals [136]. An enhancement of RSV-mediated lung eosinophilia was not seen upon challenge infection of MVA vector vaccinated animals. A parallel immunization study in infant cynomolgus macaques also suggested that vaccination with recombinant MVA did not predispose for an RSV associated immunopathology [137]. However, the combined intramuscular/intranasal immunization of these infant (<1 year old) macaques with the recombinant MVA failed to provide protection against RSV replication in the lower respiratory tract. 

To evaluate the safety and efficacy of new approaches in RSV vaccine development the infection of cattle with bovine RSV (bRSV) provides an excellent alternative model using a highly related pathogen in its natural host [138]. Recombinant MVA delivering bRSV F and G glycoprotein antigens (MVA/bRSV) were tested to protect calves against bRSV challenge [139]. Intramuscular vaccination of calves with MVA/bRSV induced bRSV specific IgG antibody and CD8^+^ T cell responses, but no detectable IgE antibodies. Upon challenge with bRSV the MVA/bRSV vaccinated calves compared to control animals demonstrated less severe lower respiratory tract symptoms, reduced pulmonary virus loads and no signs of bRSV-associated immunopathology (eosinophilic infiltrations). However, complete protection against bRSV infection or replication was not achieved. 

Overall, these previous studies suggested the safety and at least partial efficacy of first generation recombinant MVA vaccines against PIV or RSV. Other applications of recombinant MVA including mucosal delivery or the use of prime-boost strategies may contribute to further improve the effectiveness of preventive immunization against these respiratory diseases. 

### 5.2. Respiratory Diseases Caused by Emerging Coronaviruses

In the last decade, two new beta coronaviruses have been transmitted from animal reservoirs (probably bat species) to humans causing major acute respiratory diseases associated with high mortality rates, severe acute respiratory syndrome coronavirus (SARS-CoV) and, more recently, Middle East respiratory syndrome coronavirus (MERS-CoV) [140,141] (for review see [142]). At present, there are no vaccines available which are approved for emergency use in humans to prevent either SARS-CoV or MERS-CoV infections. In 2004, about one year after the recognition of SARS‑CoV in humans, recombinant MVA expressing the spike (S) protein, considered a key component of coronavirus-specific vaccines, was generated and demonstrated to elicit virus neutralizing antibodies in mice [143]. Also, another recombinant MVA vaccine producing SARS-CoV S antigen was found to induce high level (neutralizing) antibody responses in mice, rabbits and rhesus macaques [144]. Moreover, in the monkey model two immunizations with the recombinant MVA prevented replication of SARS-CoV upon respiratory challenge infection. Similarly, within the year of the appearance of MERS-CoV, a first candidate MVA vector vaccine producing the S glycoprotein of MERS-CoV (MVA-MERS-S) was obtained [145]. BALB/c mice were intramuscularly (i.m.) vaccinated with 10^8^ PFU MVA-MERS-S. Single administration of the MVA-MERS-S vaccine already induced low levels of virus-neutralizing antibodies in all animals tested. After booster immunization all vaccinated animals produced high levels of circulating antibodies that neutralized MERS-CoV. In contrast, neutralizing antibodies were not detected in sera from control animals and the specificity of the induced response was confirmed by the absence of detectable neutralization against SARS-CoV. These data support further evaluation of MVA-MERS-S as candidate emergency vaccine. In general, the swift application of recombinant MVA in response to the appearance of SARS-CoV or MERS-CoV demonstrates the suitability of this vector system to readily respond to potential threats of suddenly emerging infectious diseases.

## 6. Future Perspectives

**Figure 1:**
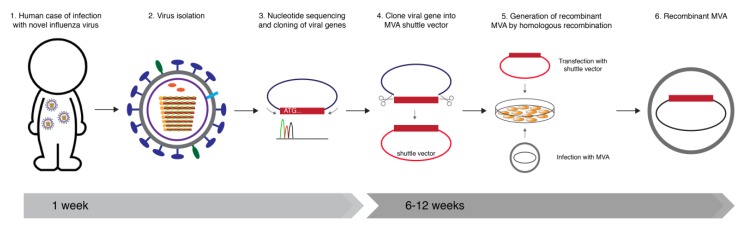
Ideal timeline for construction of an MVA-based vaccine after a human case of infection with a novel respiratory virus. Influenza virus is used as an example. (**1**) After the emergence of a novel respiratory virus with the ability of infecting humans, (**2**) the virus is isolated (**3**) and the sequence of a target gene of interest is obtained within a week. (**4**) Subsequently, the gene of interest is cloned or simply synthesized and subcloned into an MVA shuttle vector plasmid. (**5**) This shuttle vector is then transfected in cells infected with MVA. Through homologous recombination the gene of interest is inserted into the MVA genome. (**6**) By serial plaque passages on CEF, a good laboratory practice (GLP) compliant rMVA is clonally isolated . The process from cloning to obtaining the rMVA takes about 6–12 weeks.

MVA has great potential as a rapid response vaccine platform for newly emerging virus infections. Established standard protocols allow rapidly obtaining recombinant MVA (rMVA) which are suitable for clinical evaluation (for review see [146]). This can be done by infection of CEF with fully characterized non-recombinant MVA seed virus and subsequent transfection of vector plasmid DNA containing the target gene of interest. Through homologous recombination, the heterologous gene sequences are inserted in the viral genome. The rMVA is clonally selected and amplified by serial passaging on CEF derived from certified embryonated eggs of specific pathogen free (SPF) chickens. The ideal process from a human case of infection with a novel respiratory virus to the construction and isolation of a candidate rMVA takes 6–12 weeks (Figure 1). 

In order to produce enough vaccine doses for a large-scale immunization campaign, large bulks of tissue culture are required. The use of CEF is well established in vaccine manufacturing. Primary CEFs are readily prepared from embryonated eggs without need for further amplification and, as known from the production of seasonal influenza vaccines, millions of eggs can be obtained and handled within days. CEF can be produced at a large scale and cryopreserved for a later time point. However, cryopreservation impacts the quality of the cells. Therefore, especially in the context of pandemic preparedness, continuous cell lines that allow for efficient MVA propagation, such as the duck cell lines AGE1.CR and AGE1.CR.pIX [147], would be more beneficial. 

**Figure 2:**
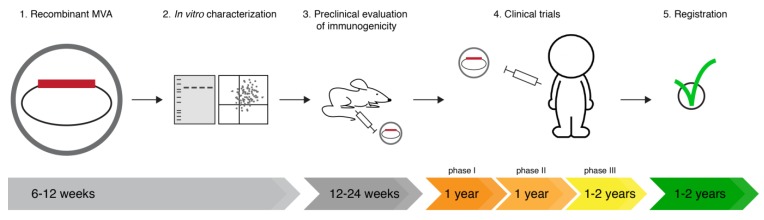
Ideal timeline for evaluation of a novel MVA-based vaccine. (**1**) A newly developed rMVA vaccine (**2**) is tested *in vitro* to assess correct gene insertion and protein expression in rMVA infected cells, e.g., by Western Blot or flow cytometry. (**3**) Subsequently, the vaccine immunogenicity and efficacy is tested in mice, ferrets and/or macaques. (**4**) If the MVA-based vaccine is successful in the pre-clinical tests, the vaccine is tested in phase I, II and III clinical trails. (**5**) Finally, when the vaccine has proven safe and effective, it can be filed for market authorization.

After generation of the rMVA, the vector vaccine needs to be validated *in vitro* to verify genetic stability, antigen expression and unimpaired growth capacity. Subsequently, *in vivo* experiments in pre-clinical models, e.g., mice, ferrets and/or macaques, are performed to determine the immunogenicity, usually testing various dosages and routes of administration, and possibly to obtain efficacy data. If successful in the pre-clinical phase, the vaccine is ready to be tested in humans. So far no MVA-based vaccine is registered and marketed for human use, but numerous vaccines are being tested in clinical trials [103]. In a phase I clinical trial, the safety of vaccine administration is tested. During phase II, safety and efficacy are further assessed, often involving various study populations. In phase III, the safety and efficacy are confirmed in large study groups. If the vaccine is successful during the different phases of the clinical trial, it can be registered for common use (Figure 2). 

However, there are some pitfalls in the development of a novel recombinant MVA vaccine, which might take precious time at the verge of a pandemic. First of all, it takes time to develop suitable animal models for newly emerging respiratory infections. Second, for each new vaccine, antigen potency and purity assays need to be developed for the appropriate quality assessment of the MVA vaccine preparations. Third, each new rMVA virus is a new biological entity. Therefore, each new vaccine must be tested thoroughly. However, in the case of a severe pandemic there would likely be no time to go through all the phases of clinical trials. Furthermore, combination vaccination strategies, e.g., priming with an adenovirus vector and boosting with an MVA vaccine, will lead to complicated regulatory procedures because two distinct biological entities need to be approved. Fourth, even though all the hands-on development work can be done rapidly, non-clinical safety testing, e.g., toxicity studies and ethics approval for animal experiments and clinical trials, and registration of the vaccine always depend on external parties, which could substantially slow down the development. Finally, also immunity to the vector, e.g., preexisting from smallpox vaccination, needs to be considered. However, studies have shown that even after multiple immunizations rMVA is still able to induce foreign antigen-specific immune responses in the presence of MVA-specific antibodies [106]. 

In addition to these practical issues, there are regulatory issues that need to be addressed. So far, only one guideline for the quality and (non-)clinical aspects of live recombinant viral vectors exists [148]. In the case of influenza, special procedures are in place in the European Union to speed up the authorization process for novel vaccines. In the case of a pandemic, the vaccine needs to become available as soon as possible. The normal procedure to approve a new vaccine takes 18–24 months, which obviously is too long for a novel vaccine in the case of influenza pandemics. Therefore, there are two procedures in place for authorization of pandemic influenza vaccines. The first one is the ‘mock-up procedure’, where a prototype vaccine is authorized. It is not possible to predict which strain will cause a pandemic. Therefore, a vaccine based on a strain that has the potential to induce a pandemic is developed and tested. This proof-of-principle vaccine is then registered. Once the viral strain that is actually causing a pandemic is identified, this can be included in the mock-up vaccine and approved quickly. Second, the ‘emergency procedure’ allows for fast-track approval of a vaccine after a pandemic already started. With such an emergency procedure, a vaccine can be approved in 70 days. MVA and other vector-based vaccines are not covered by these pandemic influenza vaccine registration procedures. So, even though a MVA-based vaccine can be developed and produced relatively quickly after a pandemic outbreak, the vaccine will not be available in time to prevent a widespread pandemic. 

## 7. Conclusions

As described, recombinant MVA has many features to serve as an excellent platform for the production of emergency vaccines. As with all vector vaccines, the foreign antigens they encode can be expressed in their native conformation, and will be authentically processed and presented to the immune system. This allows for induction of balanced humoral and cellular responses to induce solid pathogen-specific immunity. In addition, there are MVA-specific benefits. MVA is extremely replication-deficient in mammalian host cells, mediates strictly transient expression of the heterologous target genes, and the virus is highly attenuated in all *in vivo* models tested. Therefore, rMVA vaccines have an excellent safety profile considering clinical use and protection of the general environment. Furthermore, the impact of pre-existing vector immunity is limited, especially when compared to other viral vectors such as adenovirus-based vaccines [149]. Finally, rMVA vaccines are expected to be very stable over time enabling shipment to and application in remote areas with limited cold-chain maintenance.

Even though MVA-based emergency vaccines can be developed relatively quickly, there are still some hurdles to overcome. Practical issues as well as regulatory issues need to be addressed in order to develop this platform for optimal use against pandemic influenza and other (newly emerging) respiratory virus infections. 

## References

[1] World Health Organization (2014). Recommended composition of influenza virus vaccines for use in the 2014–2015 northern hemisphere influenza season.

[2] Richard M., Schrauwen E.J., de Graaf M., Bestebroer T.M., Spronken M.I., van Boheemen S., de Meulder D., Lexmond P., Linster M., Herfst S. (2013). Limited airborne transmission of H7N9 influenza A virus between ferrets. Nature.

[3] Herfst S., Schrauwen E.J., Linster M., Chutinimitkul S., de Wit E., Munster V.J., Sorrell E.M., Bestebroer T.M., Burke D.F., Smith D.J. (2012). Airborne transmission of influenza A/H5N1 virus between ferrets. Science.

[4] World Health Organization (2012). Information sheet—Observed rate of vaccine reactions—Influenza vaccine.

[5] Osterholm M.T., Kelley N.S., Sommer A., Belongia E.A. (2012). Efficacy and effectiveness of influenza vaccines: A systematic review and meta-analysis. Lancet Infect. Dis..

[6] Partridge J., Kieny M.P. (2013). Global production capacity of seasonal influenza vaccine in 2011. Vaccine.

[7] Broadbent A.J., Subbarao K. (2011). Influenza virus vaccines: Lessons from the 2009 H1N1 pandemic. Curr. Opin. Virol..

[8] He X.S., Holmes T.H., Zhang C., Mahmood K., Kemble G.W., Lewis D.B., Dekker C.L., Greenberg H.B., Arvin A.M. (2006). Cellular immune responses in children and adults receiving inactivated or live attenuated influenza vaccines. J. Virol..

[9] Forrest B.D., Pride M.W., Dunning A.J., Capeding M.R., Chotpitayasunondh T., Tam J.S., Rappaport R., Eldridge J.H., Gruber W.C. (2008). Correlation of cellular immune responses with protection against culture-confirmed influenza virus in young children. Clin. Vaccine Immunol..

[10] Krammer F., Palese P. (2013). Influenza virus hemagglutinin stalk-based antibodies and vaccines. Curr. Opin. Virol..

[11] Corti D., Suguitan A.L., Pinna D., Silacci C., Fernandez-Rodriguez B.M., Vanzetta F., Santos C., Luke C.J., Torres-Velez F.J., Temperton N.J. (2010). Heterosubtypic neutralizing antibodies are produced by individuals immunized with a seasonal influenza vaccine. J. Clin. Invest..

[12] Krause J.C., Tsibane T., Tumpey T.M., Huffman C.J., Basler C.F., Crowe J.E. (2011). A broadly neutralizing human monoclonal antibody that recognizes a conserved, novel epitope on the globular head of the influenza H1N1 virus hemagglutinin. J. Virol..

[13] Krause J.C., Tsibane T., Tumpey T.M., Huffman C.J., Albrecht R., Blum D.L., Ramos I., Fernandez-Sesma A., Edwards K.M., Garcia-Sastre A. (2012). Human monoclonal antibodies to pandemic 1957 H2N2 and pandemic 1968 H3N2 influenza viruses. J. Virol..

[14] Ekiert D.C., Kashyap A.K., Steel J., Rubrum A., Bhabha G., Khayat R., Lee J.H., Dillon M.A., O'Neil R.E., Faynboym A.M. (2012). Cross-neutralization of influenza A viruses mediated by a single antibody loop. Nature.

[15] Dreyfus C., Laursen N.S., Kwaks T., Zuijdgeest D., Khayat R., Ekiert D.C., Lee J.H., Metlagel Z., Bujny M.V., Jongeneelen M. (2012). Highly conserved protective epitopes on influenza B viruses. Science.

[16] Lee P.S., Yoshida R., Ekiert D.C., Sakai N., Suzuki Y., Takada A., Wilson I.A. (2012). Heterosubtypic antibody recognition of the influenza virus hemagglutinin receptor binding site enhanced by avidity. Proc. Natl. Acad. Sci. USA.

[17] Okuno Y., Isegawa Y., Sasao F., Ueda S. (1993). A common neutralizing epitope conserved between the hemagglutinins of influenza A virus H1 and H2 strains. J. Virol..

[18] Throsby M., van den Brink E., Jongeneelen M., Poon L.L., Alard P., Cornelissen L., Bakker A., Cox F., van Deventer E., Guan Y. (2008). Heterosubtypic neutralizing monoclonal antibodies cross-protective against H5N1 and H1N1 recovered from human IgM+ memory B cells. PLoS One.

[19] Ekiert D.C., Bhabha G., Elsliger M.A., Friesen R.H., Jongeneelen M., Throsby M., Goudsmit J., Wilson I.A. (2009). Antibody recognition of a highly conserved influenza virus epitope. Science.

[20] Margine I., Hai R., Albrecht R.A., Obermoser G., Harrod A.C., Banchereau J., Palucka K., Garcia-Sastre A., Palese P., Treanor J.J. (2013). H3N2 influenza virus infection induces broadly reactive hemagglutinin stalk antibodies in humans and mice. J. Virol..

[21] Kashyap A.K., Steel J., Oner A.F., Dillon M.A., Swale R.E., Wall K.M., Perry K.J., Faynboym A., Ilhan M., Horowitz M. (2008). Combinatorial antibody libraries from survivors of the Turkish H5N1 avian influenza outbreak reveal virus neutralization strategies. Proc. Natl. Acad. Sci. USA.

[22] Sui J., Hwang W.C., Perez S., Wei G., Aird D., Chen L.M., Santelli E., Stec B., Cadwell G., Ali M. (2009). Structural and functional bases for broad-spectrum neutralization of avian and human influenza A viruses. Nat. Struct. Mol. Biol..

[23] Friesen R.H., Koudstaal W., Koldijk M.H., Weverling G.J., Brakenhoff J.P., Lenting P.J., Stittelaar K.J., Osterhaus A.D., Kompier R., Goudsmit J. (2010). New class of monoclonal antibodies against severe influenza: Prophylactic and therapeutic efficacy in ferrets. PLoS One.

[24] Kashyap A.K., Steel J., Rubrum A., Estelles A., Briante R., Ilyushina N.A., Xu L., Swale R.E., Faynboym A.M., Foreman P.K. (2010). Protection from the 2009 H1N1 pandemic influenza by an antibody from combinatorial survivor-based libraries. PLoS Pathog..

[25] Friesen R.H., Lee P.S., Stoop E.J., Hoffman R.M., Ekiert D.C., Bhabha G., Yu W., Juraszek J., Koudstaal W., Jongeneelen M. (2014). A common solution to group 2 influenza virus neutralization. Proc. Natl. Acad. Sci. USA.

[26] Krammer F., Hai R., Yondola M., Tan G.S., Leyva-Grado V.H., Ryder A.B., Miller M.S., Rose J.K., Palese P., Garcia-Sastre A. (2014). Assessment of influenza virus hemagglutinin stalk-based immunity in ferrets. J. Virol..

[27] Hai R., Krammer F., Tan G.S., Pica N., Eggink D., Maamary J., Margine I., Albrecht R.A., Palese P. (2012). Influenza viruses expressing chimeric hemagglutinins: Globular head and stalk domains derived from different subtypes. J. Virol..

[28] Margine I., Krammer F., Hai R., Heaton N.S., Tan G.S., Andrews S.A., Runstadler J.A., Wilson P.C., Albrecht R.A., Garcia-Sastre A. (2013). Hemagglutinin stalk-based universal vaccine constructs protect against group 2 influenza A viruses. J. Virol..

[29] Goff P.H., Eggink D., Seibert C.W., Hai R., Martinez-Gil L., Krammer F., Palese P. (2013). Adjuvants and immunization strategies to induce influenza virus hemagglutinin stalk antibodies. PLoS One.

[30] Krammer F., Margine I., Hai R., Flood A., Hirsh A., Tsvetnitsky V., Chen D., Palese P. (2014). H3 stalk-based chimeric hemagglutinin influenza virus constructs protect mice from H7N9 challenge. J. Virol..

[31] Eggink D., Goff P.H., Palese P. (2014). Guiding the immune response against influenza virus hemagglutinin toward the conserved stalk domain by hyperglycosylation of the globular head domain. J. Virol..

[32] Corti D., Voss J., Gamblin S.J., Codoni G., Macagno A., Jarrossay D., Vachieri S.G., Pinna D., Minola A., Vanzetta F. (2011). A neutralizing antibody selected from plasma cells that binds to group 1 and group 2 influenza A hemagglutinins. Science.

[33] Marcelin G., Bland H.M., Negovetich N.J., Sandbulte M.R., Ellebedy A.H., Webb A.D., Griffin Y.S., DeBeauchamp J.L., McElhaney J.E., Webby R.J. (2010). Inactivated seasonal influenza vaccines increase serum antibodies to the neuraminidase of pandemic influenza A(H1N1) 2009 virus in an age-dependent manner. J. Infect. Dis..

[34] Marcelin G., DuBois R., Rubrum A., Russell C.J., McElhaney J.E., Webby R.J. (2011). A contributing role for anti-neuraminidase antibodies on immunity to pandemic H1N1 2009 influenza A virus. PLoS One.

[35] Marcelin G., Sandbulte M.R., Webby R.J. (2012). Contribution of antibody production against neuraminidase to the protection afforded by influenza vaccines. Rev. Med. Virol..

[36] Chen Z., Kadowaki S., Hagiwara Y., Yoshikawa T., Matsuo K., Kurata T., Tamura S. (2000). Cross-protection against a lethal influenza virus infection by DNA vaccine to neuraminidase. Vaccine.

[37] Sandbulte M.R., Jimenez G.S., Boon A.C., Smith L.R., Treanor J.J., Webby R.J. (2007). Cross-reactive neuraminidase antibodies afford partial protection against H5N1 in mice and are present in unexposed humans. PLoS Med..

[38] Bosch B.J., Bodewes R., de Vries R.P., Kreijtz J.H., Bartelink W., van Amerongen G., Rimmelzwaan G.F., de Haan C.A., Osterhaus A.D., Rottier P.J. (2010). Recombinant soluble, multimeric HA and NA exhibit distinctive types of protection against pandemic swine-origin 2009 A(H1N1) influenza virus infection in ferrets. J. Virol..

[39] Zebedee S.L., Lamb R.A. (1988). Influenza A virus M2 protein: Monoclonal antibody restriction of virus growth and detection of M2 in virions. J. Virol..

[40] Jegerlehner A., Schmitz N., Storni T., Bachmann M.F. (2004). Influenza A vaccine based on the extracellular domain of M2: Weak protection mediated via antibody-dependent NK cell activity. J. Immunol..

[41] Song J.M., Wang B.Z., Park K.M., van Rooijen N., Quan F.S., Kim M.C., Jin H.T., Pekosz A., Compans R.W., Kang S.M. (2011). Influenza virus-like particles containing M2 induce broadly cross protective immunity. PLoS One.

[42] El Bakkouri K., Descamps F., de Filette M., Smet A., Festjens E., Birkett A., van Rooijen N., Verbeek S., Fiers W., Saelens X. (2011). Universal vaccine based on ectodomain of matrix protein 2 of influenza A: Fc receptors and alveolar macrophages mediate protection. J. Immunol..

[43] Wu F., Huang J.H., Yuan X.Y., Huang W.S., Chen Y.H. (2007). Characterization of immunity induced by M2e of influenza virus. Vaccine.

[44] Heinen P.P., Rijsewijk F.A., de Boer-Luijtze E.A., Bianchi A.T. (2002). Vaccination of pigs with a DNA construct expressing an influenza virus M2-nucleoprotein fusion protein exacerbates disease after challenge with influenza A virus. J. Gen. Virol..

[45] Kim M.C., Lee J.S., Kwon Y.M., O E., Lee Y.J., Choi J.G., Wang B.Z., Compans R.W., Kang S.M. (2013). Multiple heterologous M2 extracellular domains presented on virus-like particles confer broader and stronger M2 immunity than live influenza A virus infection. Antivir. Res..

[46] Tompkins S.M., Zhao Z.S., Lo C.Y., Misplon J.A., Liu T., Ye Z., Hogan R.J., Wu Z., Benton K.A., Tumpey T.M., Epstein S.L. (2007). Matrix protein 2 vaccination and protection against influenza viruses, including subtype H5N1. Emerg. Infect. Dis..

[47] Neirynck S., Deroo T., Saelens X., Vanlandschoot P., Jou W.M., Fiers W. (1999). A universal influenza A vaccine based on the extracellular domain of the M2 protein. Nat. Med..

[48] Turley C.B., Rupp R.E., Johnson C., Taylor D.N., Wolfson J., Tussey L., Kavita U., Stanberry L., Shaw A. (2011). Safety and immunogenicity of a recombinant M2e-flagellin influenza vaccine (STF2.4xM2e) in healthy adults. Vaccine.

[49] Schotsaert M., de Filette M., Fiers W., Saelens X. (2009). Universal M2 ectodomain-based influenza A vaccines: Preclinical and clinical developments. Expert. Rev. Vaccines.

[50] Hillaire M.L., Rimmelzwaan G.F., Kreijtz J.H. (2013). Clearance of influenza virus infections by T cells: Risk of collateral damage?. Curr. Opin. Virol..

[51] McMichael A.J., Gotch F.M., Noble G.R., Beare P.A. (1983). Cytotoxic T-cell immunity to influenza. N. Engl. J. Med..

[52] Yewdell J.W., Bennink J.R., Smith G.L., Moss B. (1985). Influenza A virus nucleoprotein is a major target antigen for cross-reactive anti-influenza A virus cytotoxic T lymphocytes. Proc. Natl. Acad. Sci. USA.

[53] McMichael A.J., Michie C.A., Gotch F.M., Smith G.L., Moss B. (1986). Recognition of influenza A virus nucleoprotein by human cytotoxic T lymphocytes. J. Gen. Virol..

[54] Gotch F., McMichael A., Smith G., Moss B. (1987). Identification of viral molecules recognized by influenza-specific human cytotoxic T lymphocytes. J. Exp. Med..

[55] Jameson J., Cruz J., Terajima M., Ennis F.A. (1999). Human CD8+ and CD4+ T lymphocyte memory to influenza A viruses of swine and avian species. J. Immunol..

[56] Kreijtz J.H., Bodewes R., van Amerongen G., Kuiken T., Fouchier R.A., Osterhaus A.D., Rimmelzwaan G.F. (2007). Primary influenza A virus infection induces cross-protective immunity against a lethal infection with a heterosubtypic virus strain in mice. Vaccine.

[57] Kreijtz J.H., de Mutsert G., van Baalen C.A., Fouchier R.A., Osterhaus A.D., Rimmelzwaan G.F. (2008). Cross-recognition of avian H5N1 influenza virus by human cytotoxic T-lymphocyte populations directed to human influenza A virus. J. Virol..

[58] Hillaire M.L., Vogelzang-van Trierum S.E., Kreijtz J.H., de Mutsert G., Fouchier R.A., Osterhaus A.D., Rimmelzwaan G.F. (2013). Human T-cells directed to seasonal influenza A virus cross-react with 2009 pandemic influenza A (H1N1) and swine-origin triple-reassortant H3N2 influenza viruses. J. Gen. Virol..

[59] van de Sandt C.E., Kreijtz J.H., de Mutsert G., Geelhoed-Mieras M.M., Hillaire M.L., Vogelzang-van Trierum S.E., Osterhaus A.D., Fouchier R.A., Rimmelzwaan G.F. (2014). Human cytotoxic T lymphocytes directed to seasonal influenza A viruses cross-react with the newly emerging H7N9 virus. J. Virol..

[60] Hillaire M.L., Osterhaus A.D., Rimmelzwaan G.F. (2011). Induction of virus-specific cytotoxic T lymphocytes as a basis for the development of broadly protective influenza vaccines. J. Biomed. Biotechnol..

[61] Brown D.M., Dilzer A.M., Meents D.L., Swain S.L. (2006). CD4 T cell-mediated protection from lethal influenza: Perforin and antibody-mediated mechanisms give a one-two punch. J. Immunol..

[62] Teijaro J.R., Verhoeven D., Page C.A., Turner D., Farber D.L. (2010). Memory CD4 T cells direct protective responses to influenza virus in the lungs through helper-independent mechanisms. J. Virol..

[63] Alam S., Sant A.J. (2011). Infection with seasonal influenza virus elicits CD4 T cells specific for genetically conserved epitopes that can be rapidly mobilized for protective immunity to pandemic H1N1 influenza virus. J. Virol..

[64] McKinstry K.K., Strutt T.M., Kuang Y., Brown D.M., Sell S., Dutton R.W., Swain S.L. (2012). Memory CD4+ T cells protect against influenza through multiple synergizing mechanisms. J. Clin. Invest..

[65] Wilkinson T.M., Li C.K., Chui C.S., Huang A.K., Perkins M., Liebner J.C., Lambkin-Williams R., Gilbert A., Oxford J., Nicholas B. (2012). Preexisting influenza-specific CD4+ T cells correlate with disease protection against influenza challenge in humans. Nat. Med..

[66] Hillaire M.L., van Trierum S.E., Bodewes R., van Baalen C.A., van Binnendijk R.S., Koopmans M.P., Fouchier R.A., Osterhaus A.D., Rimmelzwaan G.F. (2011). Characterization of the human CD8(+) T cell response following infection with 2009 pandemic influenza H1N1 virus. J. Virol..

[67] Sridhar S., Begom S., Bermingham A., Hoschler K., Adamson W., Carman W., Bean T., Barclay W., Deeks J.J., Lalvani A. (2013). Cellular immune correlates of protection against symptomatic pandemic influenza. Nat. Med..

[68] Zheng M., Luo J., Chen Z. (2014). Development of universal influenza vaccines based on influenza virus M and NP genes. Infection.

[69] Mayr A., Munz E. (1964). Veränderung von Vaccinevirus durch Dauerpassagen in Hühnerembryofibroblastenkulturen. Zentralbl. Bakteriol. B..

[70] Mayr A., Stickl H., Müller H. (1978). Der Pockenimpfstamm MVA: Marker, genetische Struktur, Erfahrung mit der parenteralen Schutzimpfung und Verhalten im abwehrgeschwächten Organismus. Zentralbl. Bakteriol. B..

[71] Stickl H., Hochstein-Mintzel V., Mayr A. (1974). MVA-Stufenimpfung gegen Pocken. Dtsch. Med. Wochenschr..

[72] Antoine G., Scheiflinger F., Dorner F., Falkner F.G. (1998). The Complete Genomic Sequence of the Modified Vaccinia Ankara Strain: Comparison with Other Orthopoxviruses. Virology.

[73] Meisinger-Henschel C., Späth M., Lukassen S., Wolferstätter M., Kachelriess H., Baur K., Dirmeier U., Wagner M., Chaplin P., Suter M. (2010). Introduction of the Six Major Genomic Deletions of Modified Vaccinia Virus Ankara (MVA) into the Parental Vaccinia Virus Is Not Sufficient To Reproduce an MVA-Like Phenotype in Cell Culture and in Mice. J. Virol..

[74] Meyer H., Sutter G., Mayr A. (1991). Mapping of deletions in the genome of the highly attenuated vaccinia virus MVA and their influence on virulence. J. Gen. Virol..

[75] Carroll M.W., Moss B. (1997). Host Range and Cytopathogenicity of the Highly Attenuated MVA Strain of Vaccinia Virus: Propagation and Generation of Recombinant Viruses in a Nonhuman Mammalian Cell Line. Virology.

[76] Drexler I., Heller K., Wahren B., Erfle V., Sutter G. (1998). Highly attenuated modified vaccinia virus Ankara replicates in baby hamster kidney cells, a potential host for virus propagation, but not in various human transformed and primary cells. J. Gen. Virol..

[77] Sutter G., Moss B. (1992). Nonreplicating vaccinia vector efficiently expresses recombinant genes. Proc. Natl. Acad. Sci. USA.

[78] Kibler K.V., Shors T., Perkins K.B., Zeman C.C., Banaszak M.P., Biesterfeldt J., Langland J.O., Jacobs B.L. (1997). Double-stranded RNA is a trigger for apoptosis in vaccinia virus-infected cells. J. Virol..

[79] Somogyi P., Frazier J., Skinner M.A. (1993). Fowlpox Virus Host Range Restriction: Gene Expression, DNA Replication, and Morphogenesis in Nonpermissive Mammalian Cells. Virology.

[80] Tartaglia J. (1992). Highly Attenuated Poxvirus Vectors. AIDS Res. Hum. Retroviruses.

[81] Ramírez J.C., Gherardi M.M., Esteban M. (2000). Biology of Attenuated Modified Vaccinia Virus Ankara Recombinant Vector in Mice: Virus Fate and Activation of B- and T-Cell Immune Responses in Comparison with the Western Reserve Strain and Advantages as a Vaccine. J. Virol..

[82] Stittelaar K.J., Wyatt L.S., de Swart R.L., Vos H.W., Groen J., van Amerongen G., van Binnendijk R.S., Rozenblatt S., Moss B., Osterhaus A.D.M.E. (2000). Protective Immunity in Macaques Vaccinated with a Modified Vaccinia Virus Ankara-Based Measles Virus Vaccine in the Presence of Passively Acquired Antibodies. J. Virol..

[83] Veits J., Romer-Oberdorfer A., Helferich D., Durban M., Suezer Y., Sutter G., Mettenleiter T.C. (2008). Protective efficacy of several vaccines against highly pathogenic H5N1 avian influenza virus under experimental conditions. Vaccine.

[84] Werner G.T., Jentzsch U., Metzger E., Simon J. (1980). Studies on poxvirus infections in irradiated animals. Arch. Virol..

[85] Sutter G., Wyatt L.S., Foley P.L., Bennink J.R., Moss B. (1994). A recombinant vector derived from the host range-restricted and highly attenuated MVA strain of vaccinia virus stimulates protective immunity in mice to influenza virus. Vaccine.

[86] Drexler I., Staib C., Sutter G. (2004). Modified vaccinia virus Ankara as antigen delivery system: How can we best use its potential?. Curr. Opin. Biotechnol..

[87] Gasteiger G., Kastenmuller W., Ljapoci R., Sutter G., Drexler I. (2007). Cross-Priming of Cytotoxic T Cells Dictates Antigen Requisites for Modified Vaccinia Virus Ankara Vector Vaccines. J. Virol..

[88] Gorse G.J., Newman M.J., deCamp A., Hay C.M., de Rosa S.C., Noonan E., Livingston B.D., Fuchs J.D., Kalams S.A., Cassis-Ghavami F.L. (2012). DNA and Modified Vaccinia Virus Ankara Vaccines Encoding Multiple Cytotoxic and Helper T-Lymphocyte Epitopes of Human Immunodeficiency Virus Type 1 (HIV-1) Are Safe but Weakly Immunogenic in HIV-1-Uninfected, Vaccinia Virus-Naive Adults. Clin. Vaccine Immunol..

[89] Schliehe C., Bitzer A., van den Broek M., Groettrup M. (2012). Stable Antigen Is Most Effective for Eliciting CD8+ T-Cell Responses after DNA Vaccination and Infection with Recombinant Vaccinia Virus *in Vivo*. J. Virol..

[90] Blum J.S., Wearsch P.A., Cresswell P. (2013). Pathways of Antigen Processing. Annu. Rev. Immunol..

[91] Carroll M.W., Overwijk W.W., Chamberlain R.S., Rosenberg S.A., Moss B., Restifo N.P. (1997). Highly attenuated modified vaccinia virus Ankara (MVA) as an effective recombinant vector: A Murine tumor model. Vaccine.

[92] Hirsch V.M., Fuerst T.R., Sutter G., Carroll M.W., Yang L.C., Goldstein S., Piatak M., Elkins W.R., Alvord W.G., Montefiori D.C. (1996). Patterns of viral replication correlate with outcome in simian immunodeficiency virus (SIV)-infected macaques: Effect of prior immunization with a trivalent SIV vaccine in modified vaccinia virus Ankara. J. Virol..

[93] Blanchard T.J., Alcami A., Andrea P., Smith G.L. (1998). Modified vaccinia virus Ankara undergoes limited replication in human cells and lacks several immunomodulatory proteins: Implications for use as a human vaccine. J. Gen. Virol..

[94] Büttner M., Czerny C., Lehner K.H., Wertz K. (1995). Interferon induction in peripheral blood mononuclear leukocytes of man and farm animals by poxvirus vector candidates and some poxvirus constructs. Vet. Immunol. Immunopathol..

[95] Delaloye J., Roger T., Steiner-Tardivel Q.-G., Le Roy D., Knaup Reymond M., Akira S., Petrilli V., Gomez C.E., Perdiguero B., Tschopp J. (2009). Innate Immune Sensing of Modified Vaccinia Virus Ankara (MVA) Is Mediated by TLR2-TLR6, MDA-5 and the NALP3 Inflammasome. PLoS Pathog..

[96] Fleige H., Ravens S., Moschovakis G.L., Bölter J., Willenzon S., Sutter G., Häussler S., Kalinke U., Prinz I., Förster R. (2014). IL-17–induced CXCL12 recruits B cells and induces follicle formation in BALT in the absence of differentiated FDCs. J. Exp. Med..

[97] Förster R., Wolf G., Mayr A. (1994). Highly attenuated poxviruses induce functional priming of neutrophils *in vitro*. Arch. Virol..

[98] Halle S., Dujardin H.C., Bakocevic N., Fleige H., Danzer H., Willenzon S., Suezer Y., Hämmerling G., Garbi N., Sutter G. (2009). Induced bronchus-associated lymphoid tissue serves as a general priming site for T cells and is maintained by dendritic cells. J. Exp. Med..

[99] Lehmann M.H., Kastenmuller W., Kandemir J.D., Brandt F., Suezer Y., Sutter G. (2009). Modified Vaccinia Virus Ankara Triggers Chemotaxis of Monocytes and Early Respiratory Immigration of Leukocytes by Induction of CCL2 Expression. J. Virol..

[100] Waibler Z., Anzaghe M., Ludwig H., Akira S., Weiss S., Sutter G., Kalinke U. (2007). Modified Vaccinia Virus Ankara Induces Toll-Like Receptor-Independent Type I Interferon Responses. J. Virol..

[101] Gilbert S.C. (2013). Clinical development of Modified Vaccinia virus Ankara vaccines. Vaccine.

[102] Gómez C.E., Perdiguero B., García-Arriaza J., Esteban M. (2013). Clinical applications of attenuated MVA poxvirus strain. Expert Rev. Vaccine..

[103] Kreijtz J.H.C.M., Gilbert S.C., Sutter G. (2013). Poxvirus vectors. Vaccine.

[104] European Medicines Agency Authorization Procedures. http://www.ema.europa.eu/ema/index.jsp?curl=pages/special_topics/q_and_a/q_and_a_detail_000080.jsp.

[105] Saxena M., Van T.T., Baird F.J., Coloe P.J., Smooker P.M. (2013). Pre-existing immunity against vaccine vectors--friend or foe?. Microbiology.

[106] Brewoo J.N., Powell T.D., Jones J.C., Gundlach N.A., Young G.R., Chu H., Das S.C., Partidos C.D., Stinchcomb D.T., Osorio J.E. (2013). Cross-protective immunity against multiple influenza virus subtypes by a novel modified vaccinia Ankara (MVA) vectored vaccine in mice. Vaccine.

[107] World Health Organization (2014). Cumulative number of confirmed human cases for avian influenza A(H5N1) reported to WHO, 2003–2014.

[108] Imai M., Herfst S., Sorrell E.M., Schrauwen E.J., Linster M., de Graaf M., Fouchier R.A., Kawaoka Y. (2013). Transmission of influenza A/H5N1 viruses in mammals. Virus Res..

[109] Rimmelzwaan G.F., Kreijtz J.H.C.M., Suezer Y., Schwantes A., Osterhaus A.D., Sutter G. (2011). Preclinical evaluation of influenza vaccines based on replication-deficient poxvirus vector MVA. Procedia Vaccinol..

[110] Kreijtz J.H., Suezer Y., van Amerongen G., de Mutsert G., Schnierle B.S., Wood J.M., Kuiken T., Fouchier R.A., Lower J., Osterhaus A.D. (2007). Recombinant modified vaccinia virus Ankara-based vaccine induces protective immunity in mice against infection with influenza virus H5N1. J. Infect. Dis..

[111] Hessel A., Schwendinger M., Holzer G.W., Orlinger K.K., Coulibaly S., Savidis-Dacho H., Zips M.L., Crowe B.A., Kreil T.R., Ehrlich H.J. (2011). Vectors based on modified vaccinia Ankara expressing influenza H5N1 hemagglutinin induce substantial cross-clade protective immunity. PLoS One.

[112] Kreijtz J.H., Suezer Y., de Mutsert G., van den Brand J.M., van Amerongen G., Schnierle B.S., Kuiken T., Fouchier R.A., Lower J., Osterhaus A.D. (2009). Preclinical evaluation of a modified vaccinia virus Ankara (MVA)-based vaccine against influenza A/H5N1 viruses. Vaccine.

[113] Kreijtz J.H., Suezer Y., de Mutsert G., van den Brand J.M., van Amerongen G., Schnierle B.S., Kuiken T., Fouchier R.A., Lower J., Osterhaus A.D. (2009). Recombinant modified vaccinia virus Ankara expressing the hemagglutinin gene confers protection against homologous and heterologous H5N1 influenza virus infections in macaques. J. Infect. Dis..

[114] Kreijtz J.H., Suezer Y., de Mutsert G., van Amerongen G., Schwantes A., van den Brand J.M., Fouchier R.A., Lower J., Osterhaus A.D., Sutter G. (2009). MVA-based H5N1 vaccine affords cross-clade protection in mice against influenza A/H5N1 viruses at low doses and after single immunization. PLoS One.

[115] Hessel A., Schwendinger M., Fritz D., Coulibaly S., Holzer G.W., Sabarth N., Kistner O., Wodal W., Kerschbaum A., Savidis-Dacho H. (2010). A pandemic influenza H1N1 live vaccine based on modified vaccinia Ankara is highly immunogenic and protects mice in active and passive immunizations. PLoS One.

[116] Castrucci M.R., Facchini M., di Mario G., Garulli B., Sciaraffia E., Meola M., Fabiani C., de Marco M.A., Cordioli P., Siccardi A. (2014). Modified vaccinia virus Ankara expressing the hemagglutinin of pandemic (H1N1) 2009 virus induces cross-protective immunity against Eurasian 'avian-like' H1N1 swine viruses in mice. Influenza Other Respir. Viruses.

[117] Kreijtz J.H., Suzer Y., Bodewes R., Schwantes A., van Amerongen G., Verburgh R.J., de Mutsert G., van den Brand J., van Trierum S.E., Kuiken T. (2010). Evaluation of a modified vaccinia virus Ankara (MVA)-based candidate pandemic influenza A/H1N1 vaccine in the ferret model. J. Gen. Virol..

[118] Bender B.S., Rowe C.A., Taylor S.F., Wyatt L.S., Moss B., Small P.A. (1996). Oral immunization with a replication-deficient recombinant vaccinia virus protects mice against influenza. J. Virol..

[119] Berthoud T.K., Hamill M., Lillie P.J., Hwenda L., Collins K.A., Ewer K.J., Milicic A., Poyntz H.C., Lambe T., Fletcher H.A. (2011). Potent CD8+ T-cell immunogenicity in humans of a novel heterosubtypic influenza A vaccine, MVA-NP+M1. Clin. Infect. Dis..

[120] Powell T.J., Peng Y., Berthoud T.K., Blais M.E., Lillie P.J., Hill A.V., Rowland-Jones S.L., McMichael A.J., Gilbert S.C., Dong T. (2013). Examination of influenza specific T cell responses after influenza virus challenge in individuals vaccinated with MVA-NP+M1 vaccine. PLoS One.

[121] Antrobus R.D., Lillie P.J., Berthoud T.K., Spencer A.J., McLaren J.E., Ladell K., Lambe T., Milicic A., Price D.A., Hill A.V. (2012). A T cell-inducing influenza vaccine for the elderly: Safety and immunogenicity of MVA-NP+M1 in adults aged over 50 years. PLoS One.

[122] Lillie P.J., Berthoud T.K., Powell T.J., Lambe T., Mullarkey C., Spencer A.J., Hamill M., Peng Y., Blais M.E., Duncan C.J. (2012). Preliminary assessment of the efficacy of a T-cell-based influenza vaccine, MVA-NP+M1, in humans. Clin. Infect. Dis..

[123] Lambe T., Carey J.B., Li Y., Spencer A.J., van Laarhoven A., Mullarkey C.E., Vrdoljak A., Moore A.C., Gilbert S.C. (2013). Immunity against heterosubtypic influenza virus induced by adenovirus and MVA expressing nucleoprotein and matrix protein-1. Sci. Rep..

[124] Boyd A.C., Ruiz-Hernandez R., Peroval M.Y., Carson C., Balkissoon D., Staines K., Turner A.V., Hill A.V., Gilbert S.C., Butter C. (2013). Towards a universal vaccine for avian influenza: Protective efficacy of modified Vaccinia virus Ankara and Adenovirus vaccines expressing conserved influenza antigens in chickens challenged with low pathogenic avian influenza virus. Vaccine.

[125] Mullarkey C.E., Boyd A., van Laarhoven A., Lefevre E.A., Veronica Carr B., Baratelli M., Molesti E., Temperton N.J., Butter C., Charleston B. (2013). Improved adjuvanting of seasonal influenza vaccines: Preclinical studies of MVA-NP+M1 coadministration with inactivated influenza vaccine. Eur. J. Immunol..

[126] Antrobus R.D., Berthoud T.K., Mullarkey C.E., Hoschler K., Coughlan L., Zambon M., Hill A.V., Gilbert S.C. (2014). Coadministration of seasonal influenza vaccine and MVA-NP+M1 simultaneously achieves potent humoral and cell-mediated responses. Mol. Ther..

[127] Hessel A., Savidis-Dacho H., Coulibaly S., Portsmouth D., Kreil T.R., Crowe B.A., Schwendinger M.G., Pilz A., Barrett P.N., Falkner F.G. (2014). MVA vectors expressing conserved influenza proteins protect mice against lethal challenge with H5N1, H9N2 and H7N1 viruses. PLoS One.

[128] Breathnach C.C., Clark H.J., Clark R.C., Olsen C.W., Townsend H.G.G., Lunn D.P. (2006). Immunization with recombinant modified vaccinia Ankara (rMVA) constructs encoding the HA or NP gene protects ponies from equine influenza virus challenge. Vaccine.

[129] Collins P.L., Melero J.A. (2011). Progress in understanding and controlling respiratory syncytial virus: Still crazy after all these years. Virus Res..

[130] Schomacker H., Schaap-Nutt A., Collins P.L., Schmidt A.C. (2012). Pathogenesis of acute respiratory illness caused by human parainfluenza viruses. Curr. Opin. Virol..

[131] van Bleek G.M., Osterhaus A.D.M.E., de Swart R.L. (2011). RSV 2010: Recent advances in research on respiratory syncytial virus and other pneumoviruses. Vaccine.

[132] Wyatt L.S., Shors S.T., Murphy B.R., Moss B. (1996). Development of a replication-deficient recombinant vaccinia virus vaccine effective against parainfluenza virus 3 infection in an animal model. Vaccine.

[133] Durbin A.P., Cho C.J., Elkins W.R., Wyatt L.S., Moss B., Murphy B.R. (1999). Comparison of the Immunogenicity and Efficacy of a Replication-Defective Vaccinia Virus Expressing Antigens of Human Parainfluenza Virus Type 3 (HPIV3) with Those of a Live Attenuated HPIV3 Vaccine Candidate in Rhesus Monkeys Passively Immunized with PIV3 Antibodies. J. Infect. Dis..

[134] Durbin A.P., Wyatt L.S., Siew J., Moss B., Murphy B.R. (1998). The immunogenicity and efficacy of intranasally or parenterally administered replication-deficient vaccinia-parainfluenza virus type 3 recombinants in rhesus monkeys. Vaccine.

[135] Wyatt L.S., Whitehead S.S., Venanzi K.A., Murphy B.R., Moss B. (1999). Priming and boosting immunity to respiratory syncytial virus by recombinant replication-defective vaccinia virus MVA. Vaccine.

[136] Olszewska W., Suezer Y., Sutter G., Openshaw P.J.M. (2004). Protective and disease-enhancing immune responses induced by recombinant modified vaccinia Ankara (MVA) expressing respiratory syncytial virus proteins. Vaccine.

[137] De Waal L., Wyatt L.S., Yüksel S., van Amerongen G., Moss B., Niesters H.G.M., Osterhaus A.D.M.E., de Swart R.L. (2004). Vaccination of infant macaques with a recombinant modified vaccinia virus Ankara expressing the respiratory syncytial virus F and G genes does not predispose for immunopathology. Vaccine.

[138] Taylor G., Anderson L.J., Graham B.S. (2013). Bovine Model of Respiratory Syncytial Virus Infection. Challenges and Opportunities for Respiratory Syncytial Virus Vaccines.

[139] Antonis A.F.G., van der Most R.G., Suezer Y., Stockhofe-Zurwieden N., Daus F., Sutter G., Schrijver R.S. (2007). Vaccination with recombinant modified vaccinia virus Ankara expressing bovine respiratory syncytial virus (bRSV) proteins protects calves against RSV challenge. Vaccine.

[140] Drosten C., Günther S., Preiser W., van der Werf S., Brodt H.-R., Becker S., Rabenau H., Panning M., Kolesnikova L., Fouchier R.A.M. (2003). Identification of a Novel Coronavirus in Patients with Severe Acute Respiratory Syndrome. N. Engl. J. Med..

[141] Zaki A.M., van Boheemen S., Bestebroer T.M., Osterhaus A.D.M.E., Fouchier R.A.M. (2012). Isolation of a Novel Coronavirus from a Man with Pneumonia in Saudi Arabia. N. Engl. J. Med..

[142] Graham R.L., Donaldson E.F., Baric R.S. (2013). A decade after SARS: Strategies for controlling emerging coronaviruses. Nat. Rev. Micro..

[143] Bisht H., Roberts A., Vogel L., Bukreyev A., Collins P.L., Murphy B.R., Subbarao K., Moss B. (2004). Severe acute respiratory syndrome coronavirus spike protein expressed by attenuated vaccinia virus protectively immunizes mice. Proc. Natl. Acad. Sci. USA.

[144] Chen Z., Zhang L., Qin C., Ba L., Yi C.E., Zhang F., Wei Q., He T., Yu W., Yu J. (2005). Recombinant Modified Vaccinia Virus Ankara Expressing the Spike Glycoprotein of Severe Acute Respiratory Syndrome Coronavirus Induces Protective Neutralizing Antibodies Primarily Targeting the Receptor Binding Region. J. Virol..

[145] Song F., Fux R., Provacia L.B., Volz A., Eickmann M., Becker S., Osterhaus A.D.M.E., Haagmans B.L., Sutter G. (2013). Middle East Respiratory Syndrome Coronavirus Spike Protein Delivered by Modified Vaccinia Virus Ankara Efficiently Induces Virus-Neutralizing Antibodies. J. Virol..

[146] Kremer M., Volz A., Kreijtz J.H.C.M., Fux R., Lehmann M.H., Sutter G. (2012). Easy and efficient protocols for working with recombinant vaccinia virus MVA. Methods Mol. Biol..

[147] Lohr V., Rath A., Genzel Y., Jordan I., Sandig V., Reichl U. (2009). New avian suspension cell lines provide production of influenza virus and MVA in serum-free media: Studies on growth, metabolism and virus propagation. Vaccine.

[148] European Medicines Agency (2010). Guideline on quality, non-clinical and clinical aspects of live recombinant viral vectored vaccines.

[149] Harrop R., John J., Carroll M.W. (2006). Recombinant viral vectors: Cancer vaccines. Adv. Drug Deliv. Rev..

